# SARS-CoV-2 Infection in Public School District Employees Following a District-Wide Vaccination Program — Philadelphia County, Pennsylvania, March 21–April 23, 2021

**DOI:** 10.15585/mmwr.mm7030e1

**Published:** 2021-07-30

**Authors:** David Rubin, Maggie Eisen, Sophia Collins, Jeffrey W. Pennington, Xi Wang, Susan Coffin

**Affiliations:** ^1^PolicyLab, Children’s Hospital of Philadelphia, Philadelphia, Pennsylvania; ^2^Department of Pediatrics, Perelman School of Medicine, University of Pennsylvania, Philadelphia, Pennsylvania; ^3^Department of Biomedical and Health Informatics, Children’s Hospital of Philadelphia, Philadelphia, Pennsylvania; ^4^Division of Infectious Disease, Children’s Hospital of Philadelphia, Philadelphia, Pennsylvania; ^5^Division of Disease Control, Philadelphia Department of Public Health, Philadelphia, Pennsylvania.

The School District of Philadelphia reopened for in-school instruction the week of March 21, 2021, and required weekly testing for SARS-CoV-2, the virus that causes COVID-19, for all employees returning to in-school responsibilities. The resumption of in-school instruction followed a mass vaccination program using the Pfizer-BioNTech 2-dose vaccine offered under a partnership between the Philadelphia Department of Public Health and Children’s Hospital of Philadelphia to all 22,808 School District of Philadelphia employees during February 23–April 3, 2021.* The subsequent mandatory testing program provided an opportunity to assess the percentage of positive BinaxNow point-of-care antigen tests (Abbott Laboratories) identified among school staff members based on their self-reported vaccination status (i.e., received zero, 1, or 2 vaccine doses) at the time of testing. During the initial 5 weeks after schools reopened, 34,048 screening tests were performed. Overall, 0.70% of tests returned a positive result. The percentage of positive test results was lower among persons who reported receipt of 2 vaccine doses (0.09%) compared with those who reported receipt of 1 dose (1.21%) or zero doses (1.76%) (p<0.001) representing a 95% reduction in percentage of positive SARS-CoV-2 test results among persons reporting receipt of 2 compared with zero doses of Pfizer-BioNTech vaccine. Vaccination of school staff members has been highlighted as an important strategy to maximize the safety of in-person education of K–12 students this fall (*1*). These findings reinforce the importance of promoting COVID-19 vaccination among school staff members before commencement of the 2021–22 school year.

The School District of Philadelphia provided a roster of all school staff members who were invited to participate in the February 23–April 3 vaccination program. The roster included employee age, sex, and race; ethnicity of employees was not provided by the school district but was self-reported at the time of vaccination. School-based testing of all School District of Philadelphia staff members working within the district’s buildings, including those who did and did not have regular contact with students, was launched the week of March 21, 2021; mandatory weekly screening testing programs were conducted by trained nurses and designated staff members using point-of-care antigen tests. All persons reported the presence or absence of symptoms at the time of testing. Both symptomatic and asymptomatic persons who received a positive SARS-CoV-2 test result were excluded from work and required to self-isolate; asymptomatic persons were also encouraged to undergo confirmatory testing using a nucleic acid amplification test; however, no data on testing conducted outside of this school-based testing program was reported. Antigen test results were automatically reported through the secure mobile application PARapidTest (Southwest Texas Regional Advisory Council [STRAC]) to Pennsylvania’s National Electronic Disease Surveillance System.^†^

Deidentified data were analyzed to determine the percentage of SARS-CoV-2 positive test results among staff members who did not report recent exposure to a symptomatic or asymptomatic person infected with SARS-CoV-2, overall and by vaccination status. Exposure history was obtained from responses to questions asked of staff members before testing. Self-reported vaccination status was collected at the time of testing and included vaccine doses received through the county school vaccination program as well as outside the program. Because only 2-dose COVID mRNA vaccines were authorized for use by the Food and Drug Administration at the time of the study, vaccination status was categorized by self-reported number of doses of vaccine received (i.e., zero, 1, or 2 doses) at the time of testing. Information was not collected on whether ≥2 weeks had passed since receipt of the second dose, so some persons who had received 2 vaccine doses might not have been optimally protected at the time of testing.

Available data were used to calculate the percentages of staff members who were vaccinated through the program as of April 3, 2021, overall, and within each demographic group. Chi-square tests were performed to determine whether vaccination coverage of school staff members differed by demographic characteristics. Results of all school-based testing conducted during March 21–April 23, 2021, were used to calculate the percentage of asymptomatic persons with positive test results, overall and by vaccination status. Risk ratios (RRs) comparing vaccinated with unvaccinated groups were calculated. Statistical significance was set at α = 0.05 for all comparisons. All analyses were conducted using SAS software (version 9.4; SAS Institute). All data collection and analysis for the School District of Philadelphia vaccination and screening testing programs were in accordance with the authorities of local public health jurisdictions for emergency public health response activities and received a nonresearch determination.

Among 22,808 district employees eligible for COVID-19 vaccination, 10,700 (46.9%) received ≥1 dose through the program; 46.1% of staff members received both doses of vaccine through the program (Table 1). Approximately one half (51.9%) of eligible staff members aged 40–65 years were vaccinated through the program; coverage was 44.5% among persons aged 17–39 years and 27.9% among those aged >65 years. The highest coverage with ≥1 dose (95.1%) was among Asian or Pacific Islander persons, followed by White persons (65.3%). Approximately one third (32.6%) of Black or African American persons received ≥1 dose through the program. Coverage was higher among Hispanic or Latino persons (52.1%) than among persons of other ethnic groups (46.6%) and was similar among men and women.

**TABLE 1:** Characteristics of School District of Philadelphia staff members participating in a county vaccination program — Philadelphia County, Pennsylvania, February 23–April 3, 2021

Characteristic	No. of staff members eligible for vaccination	No. of staff members vaccinated through school program (%)*	p-value
**Total**	**22,808**	**10,700 (46.9)**	**NA**
**Age group, yrs**			
17–39	8,709	3,879 (44.5)	Ref
40–65	12,452	6,467 (51.9)	<0.001^†^
>65	1,170	327 (27.9)^§^	<0.001^†^
Missing	477	27 (5.6)	<0.001^†^
**Race**			
Black or African American	9,842	3,211 (32.6)	Ref
White or Caucasian	9,430	6,160 (65.3)	<0.001^†^
Asian or Pacific Islander	364	346 (95.1)	<0.001^†^
Native American	38	18 (47.4)	0.053
Other or unknown	3,134	965 (30.7)	0.056
**Ethnicity**			
Hispanic or Latino	1,232	642 (52.1)	Ref
Not Hispanic or Latino	21,576	10,058 (46.6)	<0.001^†^
**Sex^¶^**			
Female	16,501	7,762 (47.0)	Ref
Male	6,289	2,937 (46.7)	0.65

**Abbreviations:** NA = not applicable; Ref = referent group.

* Persons who received ≥1 dose of the Pfizer-BioNTech COVID-19 vaccine.

^†^ Indicates chi-square significance (p-value <0.05).

^§^ There was no mechanism to confirm receipt of vaccine via sources other than the school vaccination program. This analysis includes only persons who received COVID-19 vaccine through the school vaccination campaign.

^¶^ Excludes 18 persons for whom sex was not reported.

During March 21–April 23, the weekly number of tests performed increased from 6,215 to 12,232. By April 23, 54% of 22,808 school staff members, representing persons on-site for work responsibilities and including persons both with and without symptoms, had received 34,048 tests (Table 2), 238 (0.70%) of which were positive for SARS-CoV-2. In total, 21,083 (62%) tests were performed for persons reporting receipt of 2 vaccine doses, 1,737 (5.1%) for persons reporting receipt of 1 dose, and 11,228 (33.0%) for those who had received zero doses. Among 2-dose, 1-dose, and zero-dose vaccine recipients, 0.09%, 1.21%, and 1.76% of test results, respectively, were positive (p<0.001), representing a 95% lower percentage of positive test results among staff members who received 2 doses compared with those who were unvaccinated (RR = 0.04; 95% confidence interval [CI] = 0.02–0.07). Among staff members who did not report symptoms at the time of testing, the percentage of positive tests was lower among persons who received both vaccine doses (0.09%) than among those who had received 1 dose (0.82%) or zero doses (1.22%) (p<0.001), representing a 93% reduction in percentage of positive test results among asymptomatic school staff members who had received 2, versus zero, vaccine doses (RR = 0.07; 95% CI = 0.04–0.11). Compared with zero-dose recipients, the percentage of positive test results was 31% lower among all 1-dose recipients and 33% lower among asymptomatic 1-dose recipients; however, the differences were not statistically significant between these two groups.

**TABLE 2:** SARS-CoV-2 BinaxNow antigen tests performed during weekly testing and results received from School District of Philadelphia staff members following reopening of schools for in-person instruction, by self-reported vaccination status — Philadelphia County, Pennsylvania, March 21–April 23, 2021

Group	No. of tests performed	No. of positive results (%)	Risk ratio (95% CI)
All persons*			
Received 2 vaccine doses	21,083	19 (0.09)	0.04 (0.02–0.07)
Received 1 vaccine dose	1,737	21 (1.21)	0.69 (0.44–1.07)
Unvaccinated	11,228	198 (1.76)	Ref
**Total**	**34,048**	**238 (0.70)**	NA
**Asymptomatic, nonexposed persons**			
Received 2 vaccine doses	21,019	18 (0.09)	0.07 (0.04–0.11)
Received 1 vaccine dose	1,717	14 (0.82)	0.67 (0.39–1.16)
Unvaccinated	11,007	134 (1.22)	Ref
**Total**	**33,743**	**166 (0.49)**	NA

**Abbreviations:** CI = confidence interval; NA = not applicable; Ref = referent group.

* Includes persons who did and did not report symptoms before testing.

## Discussion

Following school reopening in Philadelphia County, Pennsylvania, on March 21, 2021, weekly point-of-care SARS-CoV-2 testing identified a 95% lower percentage of positive test results among school staff members who had received both doses of Pfizer-BioNtech vaccine compared with those among unvaccinated staff members. These results occurred when the city’s daily COVID-19 incidence was 29–33 cases per 100,000 population, and approximately 40% of strains sequenced from the region were the B.1.1.7 (Alpha) lineage.^§^ The lower percentage of positive SARS-CoV-2 test results among vaccinated staff members supports ongoing efforts to promote COVID-19 vaccination for all school employees in advance of the upcoming 2021–22 school year (*1*).

Disparities in vaccination coverage were observed, particularly among younger staff members and Black or African American persons. Targeted health education and outreach, particularly focused on these populations, might help increase vaccination coverage.

The percentage of positive test results for persons who had received 1 vaccine dose was lower than that among unvaccinated persons, but higher than that for 2-dose recipients, which underscores the importance of completing the 2-dose COVID-19 mRNA vaccination series. Current CDC recommendations indicate that fully vaccinated persons with no known exposure to COVID-19 and no COVID-19–compatible symptoms can be exempted from routine testing programs (*2*). Therefore, schools with high rates of staff member vaccination coverage might be able to implement in-person learning without the need for routine testing programs. Nevertheless, inclusion of asymptomatic vaccinated persons in routine screening programs might be necessary in settings of high levels of community transmission, particularly if vaccine escape variants or evidence of waning immunity emerge^¶^ (*3*).

The findings in this report are subject to at least six limitations. First, weekly testing possibly failed to identify persons with more significant clinical disease who were tested outside the screening program; such ascertainment bias might underestimate the impact. Second, unmeasured differences probably exist between vaccinated and unvaccinated staff members in their risk for SARS-CoV-2 exposure in community settings, incidence of previous infection, and adherence to mitigation practices (e.g., using masks and physical distancing), which might confound the findings in this study. Third, because only aggregated data from the school district’s testing program were available, it is not possible to examine the impact of vaccination on asymptomatic infection in subpopulations of school staff members. Fourth, self-report of vaccination status at the time of testing can be subject to social desirability bias that might have differentially misclassified unvaccinated persons among persons reporting vaccinations. Fifth, a higher proportion of persons reported having been vaccinated before testing than were vaccinated during the county school vaccination program; previous vaccination of staff members with other high-risk characteristics (e.g., age ≥65 years or having chronic conditions) likely contributed to this finding. Although these differences might have inflated the estimates of vaccine receipt across specific demographic groups, they would not have affected the validity of testing results or the differences in the percentage of positive test results. Finally, information was not collected on whether ≥2 weeks had passed after receipt of the second vaccine dose; thus, some persons who had received 2 vaccine doses might not have been optimally protected at the time of testing.

During a period of relatively high community transmission, weekly SARS-CoV-2 antigen screening testing of school staff members in the School District of Philadelphia, one of the country’s largest public school districts (*4*), revealed significantly fewer infections among vaccinated school staff members compared with those who were unvaccinated. Efforts to promote COVID-19 vaccination among school staff members before the upcoming 2021–22 school year will be foundational to ensure a safe learning environment (*1*).

**Summary**

**What is already known about this topic?**

Vaccination of school staff members has been highlighted as an important strategy to maximize the safety of in-person education of K–12 students this fall.

**What is added by this report?**

Weekly SARS-CoV-2 antigen screening tests required of all employees returning for in-school instruction in the School District of Philadelphia found a 95% lower percentage of positive test results among persons who reported receipt of 2 doses of COVID-19 mRNA vaccine (0.09%) than among those who were unvaccinated (1.77%).

**What are the implications for public health practice?**

Efforts to promote COVID-19 vaccination among school staff members before the upcoming 2021–22 school year will be foundational to ensure a safe learning environment.

## Footnotes

* The mass vaccination program was the principal opportunity offered within the county for school employees in the region to receive COVID-19 vaccine. Some persons aged >65 years or with chronic conditions might have obtained vaccines available to Philadelphia residents through these other priority designations, although supply at that time was scarce.

† The application did not track serial testing of individual persons, although persons who received positive test results were not retested in subsequent weeks during this study period.

§
https://www.biorxiv.org/content/10.1101/2021.04.21.440801v1

¶
https://arxiv.org/abs/2008.03283
